# Applying an Attentional Set to Perceived and Remembered Features

**DOI:** 10.1371/journal.pone.0007613

**Published:** 2009-10-29

**Authors:** Duncan Edward Astle, Anna Christina Nobre, Gaia Scerif

**Affiliations:** Department of Experimental Psychology, University of Oxford, Oxford, United Kingdom; UCL, United Kingdom

## Abstract

Previous research has examined our ability to attend selectively to particular features of perceptual objects, as well as our ability to switch from attending to one type of feature to another. This is usually done in the context of *anticipatory* attentional-set control, comparing the neural mechanisms involved as participants prepare to attend to the same stimulus feature as on the previous trial (*“task-stay”* trials) with those required as participants prepare to attend to a different stimulus feature to that previously attended (*“task-switch”* trials). We wanted to establish how participants maintain or switch attentional set *retrospectively*, as they attend to features of objects held in visual short-term memory (VSTM). We found that switching, relative to maintaining attentional set retrospectively, was associated with a performance cost, which can be reduced over time. This control process was mirrored by a large parietal and frontal amplitude difference in the event-related brain potentials (ERPs) and significant differences in global field power (GFP) between switch and stay trials. However, when taking into account the switch/stay GFP differences, thereby controlling for this difference in amplitude, we could not distinguish these trial types topographically. By contrast, we found clear topographic differences between preparing an anticipatory feature-based attentional set versus applying it retrospectively within VSTM. These complementary topographical and amplitude analyses suggested that anticipatory and retrospective set control recruited a qualitatively different configuration of underlying neural generators. In contrast, switch/stay differences were largely quantitative, with them differing primarily in terms of amplitude rather than topography.

## Introduction

When we process perceptual input we do so in the context of an attentional set. For example, when shopping for red apples we might distinguish them from other fruit on the basis of their color or shape. Our ability to apply an attentional set, enhancing task-relevant perceptual information and/or suppressing task-irrelevant information, is well documented, as is our ability to change from one attentional set to another. However, our ability to apply an attentional set *retrospectively*, to information stored in visual short term memory (VSTM), is only beginning to be explored.

In a now classical study, Sperling [1] presented participants with an array of items to be later recalled (e.g., variably coloured letters). Following their disappearance, he cued participants' attention to a subset of the items (for example, by directing them to attend to a specific row of items within the memory array). When the delay between the array and the cue was very brief, the partial report condition resulted in improved recall for the cued row compared to the condition when participants were required to report the array in full. Although the experiment was not designed with attentional orienting effects in mind, benefits suggest that information in memory can be successfully selected on the bases of cues and biased for efficient recall. More recently studies have explored the mechanisms by which we create internal representations in VSTM, and selectively modulate them via attentional mechanisms after the offset of perceptual information, according to our task goals [e.g.], [ 2]–[9]. This implies that VSTM is not simply a passive store of information, but rather that information held in this way can be manipulated according to top-down biases directly related to task-relevant goals, just as has been proposed for incoming perceptual input [10]. Event-related brain potentials (ERPs) have been used to chart the temporal dynamics of these processes. To date these studies have primarily looked at the biasing of a particular spatial location within a VSTM representation: for example, Griffin and Nobre [3] found both similarities and differences in the way spatial attentional biases are applied to incoming perceptual input and representations held in VSTM; lateralised components locked to the onset of spatially informative attention cues were similar for prospective and retrospective spatial attentional orienting. An early positive non-lateralised potential over frontal scalp and a later increased relative positivity over posterior scalp regions, coupled with an increased negativity over the anterior scalp, set the deployment of attention to perceptual versus remembered input apart. By contrast to studies that provide participants with retrospective *spatial* cues (such as Sperling's cueing of a line of letters), the current study explores the biasing of task-relevant *features* (i.e. colors and shapes) within perceived and stored representations.

We designed a novel paradigm in which, rather than selecting particular locations within remembered arrays, participants had to select the particular *features* within remembered arrays. Whilst a number of studies have examined the selection of particular remembered objects based upon their locations [e.g. 11], [12], there have been very few examining the selection of remembered objects based upon their features. One previous electrophysiological study did compare the selection of perceived and remembered objects on this basis, however [2]. In this study participants selected particular objects because they contained probe-matching task-*relevant* features, and ignored/suppressed other objects because they contained probe-matching task-*irrelevant* features. Feature-selection and feature-suppression elicited contrasting spatially-specific electrophysiological effects for task-relevant and irrelevant features, both when searching perceived and remembered objects. These effects suggest that participants were able to use attentional mechanisms to differentiate features on the basis of task relevance, whether they were perceptually present or stored in VSTM. However, the polarity of these effects were both reversed when searching remembered objects, relative to searching perceived objects, implying that feature-based selection in memory and perception might not proceed via identical mechanisms. For this reason, the current study explored the mechanisms by which an attentional set is controlled when it pertains to features within perceived objects and within remembered objects. In short, we wanted to directly compare *anticipatory* attentional set control, with *retrospective* attentional set control.

Attentional set control is often studied using a task-switching paradigm [13]–[15]. A typical paradigm is to present participants with a cue indicating that they should attend to a specific feature of the upcoming stimulus, the upcoming stimulus then appears, the participant attends selectively to the task-relevant feature, and selects a response accordingly. In such studies participants typically apply the same stimulus-response mapping on every trial, but on a subset of trials switch from attending to one stimulus feature (e.g. color) to attending to another stimulus feature (e.g. shape). Because this process is necessarily rapid, methods with a high temporal resolution, such as ERPs, are particularly useful for studying it [16]. ERPs elicited by cues appearing in advance of a switch of task are usually compared with those indicating a repeat of task, with the aim of capturing those rapid cue-locked processes that enable participants to switch tasks. To our knowledge there have been very few electrophysiology studies directly comparing attentional set switching with the more standard intentional set switching (when the attended feature is constant but the stimulus-response mapping changes across trials). Across two studies Rushworth and colleagues [14], [15] did just this. In both cases participants elicited a parietal positivity of greater amplitude on switch relative to stay trials, with the same underlying dipole explaining the effect in both attentional and intentional set switching. On this basis they suggested that the cue-locked parietal positivity is likely an index of some basic switching mechanism, common to both switching attentional set and intentional set. Consistent with this suggestion, this parietal positivity is the most robustly reproduced effect across different types of set switching studies. It has been variably labelled the differential positivity [D-Pos, 17]–[19]; PP-AN [20]; and the Late Parietal Positivity [LPP, 21]–[27] emerging around 300 milliseconds post cue. On some occasions this is accompanied by an increased cue-locked negativity over the frontal electrodes for switch trials, with a similar timing to the parietal positivity [20], [22], [23]. Many have labelled either one or both of these differences as markers of *‘task-set reconfiguration’* [TSR, 17], [19], [20], a mechanism akin to a ‘mental gear change’, which necessarily precedes task-specific processing [28]. It is proposed that this reconfiguration of set takes time, resulting in a performance cost that is routinely observed for switch trials relative to stay trials – termed the ‘switch-cost’. The TSR account has characterised the view that the switch-cost results from some active time-consuming process, which occurs on switch but not stay trials [29], rather than from the passive dissipation of the previously used task-set [30]. It has been suggested that because the anticipatory parietal positivity mirrors a reduction in switch-cost with preparation, it is likely to index some TSR-like process [e.g. 17].

In order to explore attentional set control within perception and memory, we combined aspects of the traditional set switching paradigm with those of a VSTM paradigm. Like a number of VSTM studies, on each trial participants were presented with a to-be-remembered array of objects (colored shapes), and subsequently had to make some judgement about a probe stimulus – in this case the location of the probe's task-relevant feature in the preceding memory-array. As is the case in traditional attentional set switching studies, on any given trial participants should either attend to the color or the shape of the array objects, and match the probe according to either its shape or color; on some trials participants will have to switch from attending to shape to attending to color or vice versa (switch trials); on some trials participants will attend to the same feature dimension that they attended to previously (stay trials). We presented cues either prior to the onset of the memory-array, in which case participants could prepare an attentional set in anticipation of incoming perceptual input, or after the memory-array, in which case participants would initially have to remember all features in the memory-array, and apply an attentional set retrospectively to that stored representation. Using these ‘pro-cue’ and ‘retro-cue’ trials, respectively, we were able, for the first time, to compare anticipatory and retrospective attentional set control.

There are a number of reasons to expect differences in the mechanisms governing anticipatory and retrospective attentional set control. It is worth noting these at the outset. By definition, retro-cues have a particular advantage over pro-cues in one respect: retro-cues enable participants to selectively attend to the *specific* features of the memory-array objects that they have just processed, essentially enabling them to prepare the specific stimulus-response mapping necessary for that trial (e.g. “red object on the left and green object on the right”). Traditional pro-cues will enable participants to prepare an attentional set at an abstract level (e.g. “attend to each objects' color”), but they will have to wait until after the onset of the memory-array to fully specify the stimulus-response mapping for that trial. Conversely, by definition, pro-cues will have a particular advantage over retro-cues in one respect: pro-cues enable participants to attend to and retain task-relevant features of the memory-array objects, filtering out task-irrelevant features. Retro-cue trials will require participants to initially retain both task-relevant and task-irrelevant features, with them having to wait until the onset of the retro-cue in order to select the relevant features for that trial. In short, retro-cues will require participants to retain multi-feature objects from which they will subsequently select the relevant features, whereas pro-cues will enable participants to retain only relevant features in the first instance.

## Methods

### Main experiment

#### Participants

Eighteen participants participated in the experiment. One was excluded on the basis of their behavioural performance, with performance being no better than chance in some conditions. A further three participants were excluded from the ERP analyses because of excessive ocular artefacts. The participants comprised seven females and were an average of 25.35 years old (±2.82 std.dev). All participants provided written informed consent and were paid for their participation. The study was approved by the Central University Research Ethics Committee at the University of Oxford, UK.

#### Task

**Figure 1:**
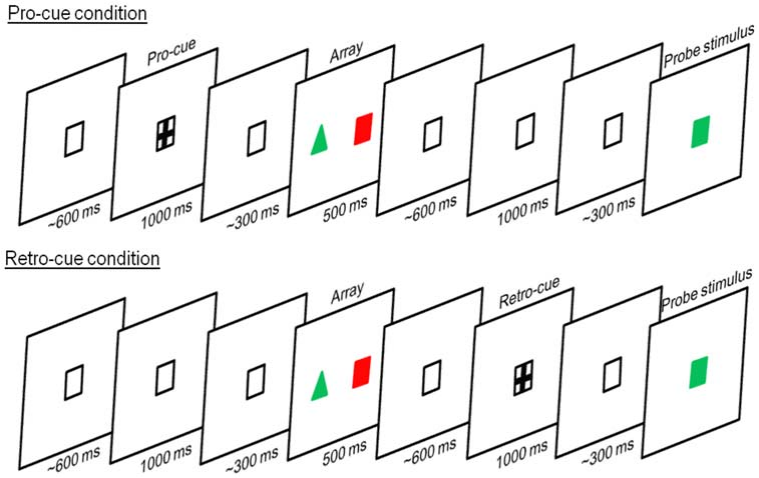
Trial order schematic. A trial order schematic, showing the timing of pro- and retro-cues, array and probe stimuli.

In order to rule out the contribution of response selection to our measures of task set, the process of applying a set and the selection of a response were separated by modifying the traditional task-switching paradigm. Participants deployed their attentional set to an array of items (two differently colored shapes) but would not know the correct feature upon which to base the response until the onset of a probe (a single colored shape) at the end of each trial (see Figure 1). On half of the trials we cued participants in advance of the array, as is traditionally done in paradigms of this sort [e.g. 14], [15], with these being termed pro-cue trials, which could be either switch or stay. On the other half of trials we cued participants *after* the array had disappeared, but before the probe appeared, with these being termed retro-cue trials; again these could be either switch or stay.

Participants were instructed to match the probe stimulus to the preceding array of two colored shapes. This matching was done either on the basis of the color of the probe and array items, or on the basis of their shape. We termed these the ‘color’ and ‘shape’ tasks. Participants responded by pressing the response button on the side that corresponded to the location of the matching feature in the preceding array. The color of the probe would always match with one of the array items, as would its shape. On half of trials the probe would match the color feature of one item and the shape feature of the other item in the array, meaning that the tasks would be incongruent to one another. On the other half of trials the probe would match both the color and shape features of one item, with the other array item being a complete non-match, meaning that the tasks were congruent with one another. The stimulus-response mappings changed on a trial-by-trial basis, with them being defined on every trial as the location (left versus right) of the task-relevant stimulus features in the memory-array. The “match to shape” and “match to color” tasks occurred in a random order and with equal frequency. Thus on half of all trials participants switched from performing one task to performing the other (‘switch trials’), whereas on the other half of trials they repeated the previously performed task (‘stay trials’). Responses were made on a two-button mouse using the left- and right-hand index fingers.

At the start of each block participants were instructed as to which task they should perform on the first trial of that block. For each subsequent trial, participants were presented with a cue, indicating that they should either ‘stay’ doing the same task or ‘switch’ to performing the alternative task. These cues took the form of a ‘+’ or an ‘×’, and their meaning was counterbalanced across participants. This instructive cue appeared either prior to the onset of the stimulus array, in which case it was termed a ‘pro-cue’, or following the stimulus array, in which case it was termed a ‘retro-cue’ (see Figure 1). No instructive information was given with either the array or probe stimuli, making the cue the only means of discerning which features were relevant for the current trial. The best means of cueing participants is certainly debatable, with different researchers opting to use different types of cue. Like a number of previous studies [e.g. 14], [15], [22], [23], we used ‘switch’ and ‘stay’ transition cues, rather than more conventional ‘shape’ and ‘color’ task cues, such that cue-change did not confound task-change [31]. That is, a change in cue is as likely to result in a repeat of task as it is in a change of task. However, this choice of cue introduces additional control processes: relative to a conventional cue, there will be a greater need to update and retain which class of feature was relevant on the immediately preceding trial; otherwise participants will not know which feature-set to repeat, or which feature-set to switch to. This may contribute equally to performance on both switch and stay trials, and may therefore mask any subtle behavioural switch-stay differences.

#### Design

All blocks of trials throughout the experiment comprised only 10 trials, and participants were able to keep track of which task they should be performing. Data from the first trial of each block were discarded. The experiment was preceded by a short familiarisation and training phase. Participants performed one pure block of trials for each of the two tasks (the order of which was counterbalanced across participants). This was followed by four blocks of mixed trials, for which participants were given feedback on each trial. Following this phase, participants proceeded to 40 blocks of experimental trials. Half of these blocks used pro-cues, and the other half used retro-cues. Cue-type was blocked because it would become very confusing for participants to switch between different tasks as well as between pro-cue and retro-cue trials. There were five consecutive blocks of each, followed by five blocks of the other cue-type and so on, to avoid order effects. The order of these blocks was also counterbalanced across participants. No feedback was given for the experimental trials.

All trials followed the same pattern: following the response to the previous probe, an interval of 1900 ms preceded the next array onset; the array was presented on the screen for 500 ms. Following another interval of 1900 ms, the probe was presented (Figure 1). The probe stimulus remained on the screen until participants made a response, at which point the next trial started. On pro-cue trials the pre-array interval included a cue for 1000 ms, the onset of which was jittered by 200 ms around 600 ms post-response. A short interval (300 ms, jittered around 200 ms) followed the offset of the cue until the onset of the array. A blank interval followed the array for 1900 ms until the onset of the probe. On retro-cue trials, the trial started with a blank interval for 1900 ms, until the onset of the array. The post-array interval included a cue for 1000 ms, the onset of which was also jittered by 200 ms around 600 ms post array. This was followed by a short interval (300 ms, jittered around 200 ms) until the onset of the probe. Occasionally (two trials per block), the cues appeared late (1600 ms post response/array) and only briefly (200 ms), leaving participants only 300 ms to prepare their attentional set between the onset of the array/probe. We varied the cue intervals in this way in order to examine the preparation effect – the reduction of switch-costs with increasing cue interval, described in the Introduction section – as this is usually taken as evidence for some TSR-like process. On retro-cue trials, this lack of preparation would be captured by performance to the probe; it will be important to compare switch-costs across the two cue intervals, particularly on retro-cue trials, in order to evidence this preparation effect. Pro-cues ought not to capture this preparation effect, as participants would have the duration of post-array interval (1900 ms), to engage in any process that could not be completed before the array, before having to make a response. Nonetheless we manipulated the cue interval in both pro- and retro-cue conditions in the same way, thus avoiding any confound of cue-onset predictability and cue-type.

Previous research has shown that these design features (short CTI “catch” trials, short blocks of ten trials, transition- rather than task-cues, instructive information only with the cue) provide participants with the circumstances and/or incentive to prepare fully the task-set in advance of the imperative stimulus [22].

#### Stimuli

Throughout each trial, until the onset of the probe, there was a small white square (∼0.8°×0.8°) present at fixation. When the cue was present, a black ‘+’ or ‘×’ appeared within this square. The two cues were identical in shape and size, but rotated through 45° to form either a ‘+’ or an ‘×’. The array took the form of two large shapes (vertical: ∼3.1°× horizontal: ∼2.1°) which were presented simultaneously, with the inner edge of each shape falling at ∼2.9° to the left and right of fixation. At the end of each trial, the probe appeared. This appeared at fixation and was ∼1.2°×1.2° in size. The shapes used in the arrays and for the probe stimulus were a triangle and a square, which were either red or green.

#### EEG acquisition and preprocessing

The EEG was recorded continuously using NuAmps amplifiers (Neuroscan, Inc.) from 40 silver/silver chloride electrodes placed on the scalp with an elasticated cap, positioned according to the 10–20 international system. The montage included six midline sites (FZ, FCZ, CZ, CPZ, PZ, and OZ) and 14 sites over each hemisphere (FP1/FP2, F3/F4, F7/F8, FC3/FC4, FT7/FT8, C3/C4, T7/T8, CP3/CP4, TP7/TP8, P3/P4, P7/P8, PO3/PO4, PO7/PO8, and O1/O2). Electrodes were placed around the eyes to monitor for blinks and eye movements. Additional electrodes were used as ground and reference sites. Electrodes were referenced to the right mastoid site during recording. The electrode between FPZ and FZ on the midline served as the ground electrode. Electrode impedances were kept below 5 kΩ. The ongoing brain activity at each electrode site was sampled every 1 ms (1000 Hz analogue-to-digital sampling rate). Activity was filtered with a low-pass filter of 300 Hz. The EEG was recorded continuously during the entire duration of each experimental run.

The data were subsequently re-referenced to the average of the montage electrodes, and a 40-Hz low pass filter was used. Epochs were formed from 50 ms before the onset of the cue, to 1000 ms post-cue. Pro-cues were necessarily closer in time to the previous response than retro-cues, and the pre-stimulus baseline was therefore more likely to be contaminated by any response artifacts, such as eye blinks that accompany responses, than it would be for retro-cues. A baseline period of 50 ms either side of the cue onset was therefore used. This baseline ensured that switch and repeat waveforms were equal at cue onset, for both pro- and retro-cues. This strict baseline eliminated any lingering effects that might have occurred before the onset of the cue, and ensured that any between-cue differences we observed were genuine, and not simply the result of a difference in the baseline period. This produced four waveforms: pro-cue stay, pro-cue switch, retro-cue stay and retro-cue switch. Eye movements and blinks (±50 µv in either EOG channel) were removed prior to averaging. This process was checked manually.

#### ERP analyses

Only data taken from the long cue intervals were submitted to ERP analyses, hence them being heavily weighted in the design. We removed from further analyses any trials upon which subjects made an error, although we did not remove slow responses (those >2 std. dev. greater than each subjects mean), but these were a very small proportion of the overall trials included. Long CTI trials were selected in order to capture those processes of active preparation, following both the pro-cues and the retro-cues. The short cue interval trials were only included to provide a behavioural comparison with the long cue interval trials, because they would demonstrate participants' behaviour when they had not time to engage in active preparation to the same extent. We compared identical cue-locked epochs (0–1000 ms), across the four trial types (pro-cue task-stay, pro-cue task-switch, retro-cue task-stay, and retro-cue task-switch) using both topographical and amplitude analyses.

#### Topographical analysis

Our analysis focussed first on comparing topographies across and within conditions. A number of previous ERP studies of set-shifting have compared the amplitude of effects, contrasting switch and stay trials on this basis [e.g. 18], [19], [21]–[27]. Whilst this approach has proved to be a good means of exploring the mechanisms of set control, there are inherent limitations: i) the data tend to be massively reduced, with most researchers choosing specific electrodes, time points and/or peaks – a process which introduces a large amount of experimenter bias; ii) this approach also requires a number of a priori assumptions, such as the importance of certain peaks; iii) the reference electrode will always remain the choice of the researcher, and could affect the result of any analysis; and, most critically, iv) analysing the amplitude alone can make it particularly difficult to distinguish quantitative changes (i.e. a change in amplitude, with no change in neural generators) from qualitative changes (i.e. a change in the configuration of neural generators). By contrast, a significant difference in the *distribution* of ERP effects for distinct trial types, provided that the distributions are strictly normalised, can only result from different neural generators or their different weightings [32]–[35]. Thus, whilst much can be learnt by comparing the amplitude of switch and stay ERP effects, if one wants to compare the neural generators that underpin these effects, one must analyse normalised topographies. A recent paper has taken this approach [36], providing an additional means by which one can compare switch and stay trials. Our topographical analysis protocol largely follows this previous paper.

We used the software CarTool (http://brainmapping.unige.ch/Cartool.htm) to normalise topographies from our four conditions by global field power (GFP) – the root mean squared of the voltage across the average-referenced electrodes – and to perform all topographical analyses. Each of our four conditions was first treated at a group-average level, and topographies were fitted to each grand-average waveform. Initially each sample (one per millisecond) was expressed as a topography. For our purposes topographies with greater than 0.97 correlation were clustered together, both within a condition and across the four conditions. In addition, we specified that no single cluster should persist for less than 20 ms. An iterative procedure was then applied to these clusters of topography, known as *Atomize and Agglomerate Hierarchical Clustering*, whereby the “worst” cluster (i.e. that with the lowest explained variance) was broken up into its constituent maps. These free maps were then independently reassigned to the cluster with which they correlated most highly. After this process, one is left with a series of possible solutions to the data. We selected the optimal solution on the basis of the *Cross-Validation criterion*. This measure of residual variance provides the optimum number of clustered topographies to explain the variance in topographical distribution, much like a latent-variable analysis of behavioural data would. Thus at the end of this process we obtained a series of larger clusters, akin to periods of stable topography within each condition, with the topography for each cluster being defined as the mathematical average of the topographies in that cluster. This process is referred to as ‘group-level segmentation’.

We examined any between-condition differences in topography at a within-subject level with two statistical tests. The first of these is referred to as a T-Anova, and is intended to test whether two topographies are sufficiently different to be statistically distinct. This compares the global dissimilarity between the topographical distributions of two conditions [37]. Again we submitted normalised data to this calculation, comparing the conditions that our group segmentation suggested to be different, across the time windows identified in the segmentation. Given that global dissimilarity is a unidirectional measure, results of the T-Anova were deemed significant if they reached a one-tailed significance level (p<0.05). Where this confirmed the between-condition differences that we observed in the group-average result, we tested these differences using a second statistical technique, referred to as *within-subject fitting*, with the aim of testing whether these distinct distributions were present to reliably different extents between the experimental conditions. This compared the frequency of the different candidate maps within that time period, for each of the conditions, in a competitive way. To do this we compared the number of time points for which that topography provided a higher spatial correlation than the other candidate topography/ies, with the specified time window. We analysed these values with a repeated-measures ANOVA. Whilst the T-Anova informed us about whether the topographies across conditions differed, it was the *within-subject fitting* process that fully tested whether different candidate topographies, identified by the group-segmentation, accounted for different conditions (see 33 for a tutorial review).

#### Amplitude analysis

Because the segmentation and fitting procedures focussed on normalised topographical maps, this analysis is purposefully insensitive to any differences in the overall amplitude of effects. For this reason, we also performed an analysis on the un-scaled ERPs, which would identify any amplitude differences that might exist in the absence of any significant topographical differences. We compared voltages over 40-ms time bins throughout the epochs, using fifteen electrodes, with five midline sites (Fz, FCz, Cz, CPz, and Pz), and five pairs of corresponding lateralised sites (F3/F4, FC3/FC4, C3/C4, CP3/CP4, P3/P4). We conducted an ANOVA at each 40-ms bin, with the within-participants factors of cue-type (pro-cue versus retro-cue trial), task-switching (stay versus switch trial), electrode position along the anteroposterior axis (five levels) and electrode position along the lateral axis (three levels). We only counted effects as being genuinely significant if they persisted for two consecutive bins [see also 14], [15]. All of the results from the ANOVAs are reported using the Greenhouse-Geisser correction, to account for the potential non-sphericity of ERP data [38].

We also compared directly the cue-locked GFP across the four trial types. This provides the perfect complementary approach to the topographical analysis: whilst the topographical analysis controls for differences in the strength of an effect and identifies changes in the distribution of an effect, the GFP comparison controls completely for differences in topography and instead compares differences in the strength of an effect. We compared the GFP across 50 ms bins, using a repeated-measures ANOVA, with the factors of cue-type and switch/repeat.

### Supplementary behavioural experiment

In addition to the behavioural data taken from the main experiment, we ran a supplementary behavioural experiment. This used only retrocues, and had a greater number of trials than the main experiment. The purpose of this supplementary experiment was to test further whether retro-cues elicited some time-consuming switch process.

#### Subjects

Sixteen participants participated in Experiment 2, none of whom had performed Experiment 1. Two were excluded on the basis of their behavioural performance, with neither performing better than chance in some conditions. The remaining fourteen subjects comprised 10 females and were an average of 23.73 years old (±3.69 std.dev). All subjects provided written informed consent and were paid for their participation. The study was approved by the medical ethics review board at the University of Oxford, UK.

#### Design

The task and stimuli were identical to those used in the main experiment. In this supplementary experiment we only used retro-cue trials, with half of all trials having the exact same timing as in the retro-cue trials in the main experiment, and the other half having an additional 2000 ms between the offset of the array and the onset of the cue. We varied the cue-probe interval in the same way as we had in the main experiment: On retro-cue trials without the extra delay the cue appeared for 1000 ms, the onset of which was jittered by 200 ms around 600 ms post array. Occasionally (two trials per block), the cues appeared late (1600 ms post array) and only briefly (200 ms). On retro-cue trials with the delay the cue appeared for 1000 ms, the onset of which was jittered by 200 ms around 2600 ms post array. Occasionally (two trials per block), the cues appeared later (3600 ms array) and only briefly (200 ms). In summary, in this second experiment we manipulated event timings in two ways: the overall delay of the probe after the array, and the delay of the probe after the cue (the ‘CTI’). These two manipulations were orthogonal to one another.

## Results

### Main experiment behavioural findings

We only analysed reaction-time (RT) data from accurate trials. These data were trimmed to remove RTs that were over two standard deviations above each participant's mean RT, separately for each condition. The trimmed mean RTs were then submitted to a repeated-measures ANOVA, with the within-participants factors of cue-type, switch/stay and CTI. The three-way interaction was non-significant [F(1,16) = 2.81, p = 0.115]. There were main effects of both CTI [F(1,16) = 100.63, p<0.001], with short CTI trials having slower RTs than long CTI trials [830 ms versus 642 ms, respectively], and of cue-type [F(1,16) = 96.45, p<0.001], with retro-cues having longer RTs than pro-cues [852 versus 619 ms, respectively]. CTI and cue-type interacted [F(1,16) = 100.63, p<0.001], resulting from significantly slower RTs on short CTI than on long CTI retro-cue trials [short CTI: 1045 ms; long CTI: 670 ms. F(1,16) = 140.12, p<0.001], whereas there was no such effect of CTI on pro-cue RTs [short CTI: 615 ms; long CTI: 624 ms. F(1,16) = 0.26, p = 0.617]. There was no main effect of switching, or any interactions between switching and any other factor. We looked specifically for an interaction between CTI and switch/stay on retro-cue trials. As was outlined in the Methods section, we reasoned that this was the best opportunity to observe the traditional preparation effect. There was a significant two-way interaction between CTI and switch/stay [F(1,16) = 4.662, p = 0.046]. This resulted from responses on short CTI switch trials being slower than short CTI stay trials [1070 versus 1020 ms, respectively, F(1,16) = 3.503, p = 0.080], whereas responses on long CTI switch trials were slightly faster than long CTI stay trials [646 ms versus 673 ms, respectively, F(1,16) = 2.178, p = 0.159].

We conducted the same analysis on the error data. Again there was no three-way interaction [F(1,16) = 0.475, p = 0.501]. As with the RT data, there was a main effect of cue-type [F(1,16) = 16.01, p = 0.001], which interacted with CTI [F(1,16) = 5.42, p = 0.033], resulting from no difference in error rates on pro-cue trials [CTI 200, 4.7%; CTI 1000, 6.1%; F(1,16) = 1.28, p = 0.275], but a significant difference on retro-cue trials [CTI 200, 15.5 %; CTI 1000, 11.4%; F(1,16) = 5.42, p = 0.033]. There was no main effect of CTI [F(1,16) = 0.2.241, p = 0.154], or task-switching [F(1,16) = 0.013, p = 0.911], or any interaction between task-switching and any other factor. There was no evidence of the preparation effect in the retro-cue condition, where we would have most likely observed it; the switch/stay difference was not significantly bigger at the short CTI [16% versus 15%, respectively], than at the long CTI [10.9% versus 12%, respectively, F(1,16) = 0.116, p = 0.738].

### Supplementary behavioural experiment

The behavioural data from the main experiment can be taken as evidence for some time-consuming switch process on retro-cue trials; participants showed a relative switch-cost of 50 ms on short CTI trials, and a relative switch-benefit of 27 ms on long CTI trials. We analysed the data from the supplementary experiment to evidence further this preparation effect, in a design with a far great number of retro-cue trials.

We processed the data in the same way as in the main experiment. The mean RTs from the trimmed data were then submitted to a 3-way ANOVA, with the within subjects factors of delay/no delay, CTI and switch/stay. There was no three-way interaction [F(1,13) = 2.589, p = 0.132]. The only two way-interaction that we observed was between CTI and switch/stay [F(1,13) = 16.868, p = 0.001], confirming the effect on the retro-cue trials from the main experiment. This resulted from a significant switch-cost at the short CTI [switch: 1000 ms; stay: 910 ms. F(1,13) = 8.104, p = 0.014], but a significant switch-related benefit at the long CTI [switch: 655; stay: 692. F(1,13) = 5.250, p = 0.038]. We made the same comparison with the error data. There was no three-way interaction [F(1,13) = 0.156, p = 0.700]. The only effect that reached significance in the error data was a main effect of CTI, with performance being more error-prone on the short CTI trials [short CTI: 15% errors; long CTI: 10% errors. F(1,13) = 13.914, p = 0.003].

The results of the supplementary experiment show the same pattern as the behavioural data from the main experiment. The particular comparison of interest – retro-cue switch versus retro-cue stay at the long and short CTI – has far greater power in the supplementary experiment, with there being twice as many trials as in the main experiment, hence it being reported here. On the basis of these data, in addition to the behavioural data from the main experiment, we concluded that switching attentional set retrospectively does involve some time-consuming switch process, which can be fully overcome with preparation. It seems reasonable to assume that the same is true of switching attentional set prospectively [e.g. 15], but there is no way of verifying this with our paradigm.

### Main experiment cue-locked ERP results

#### Group-level segmentation

**Figure 2:**
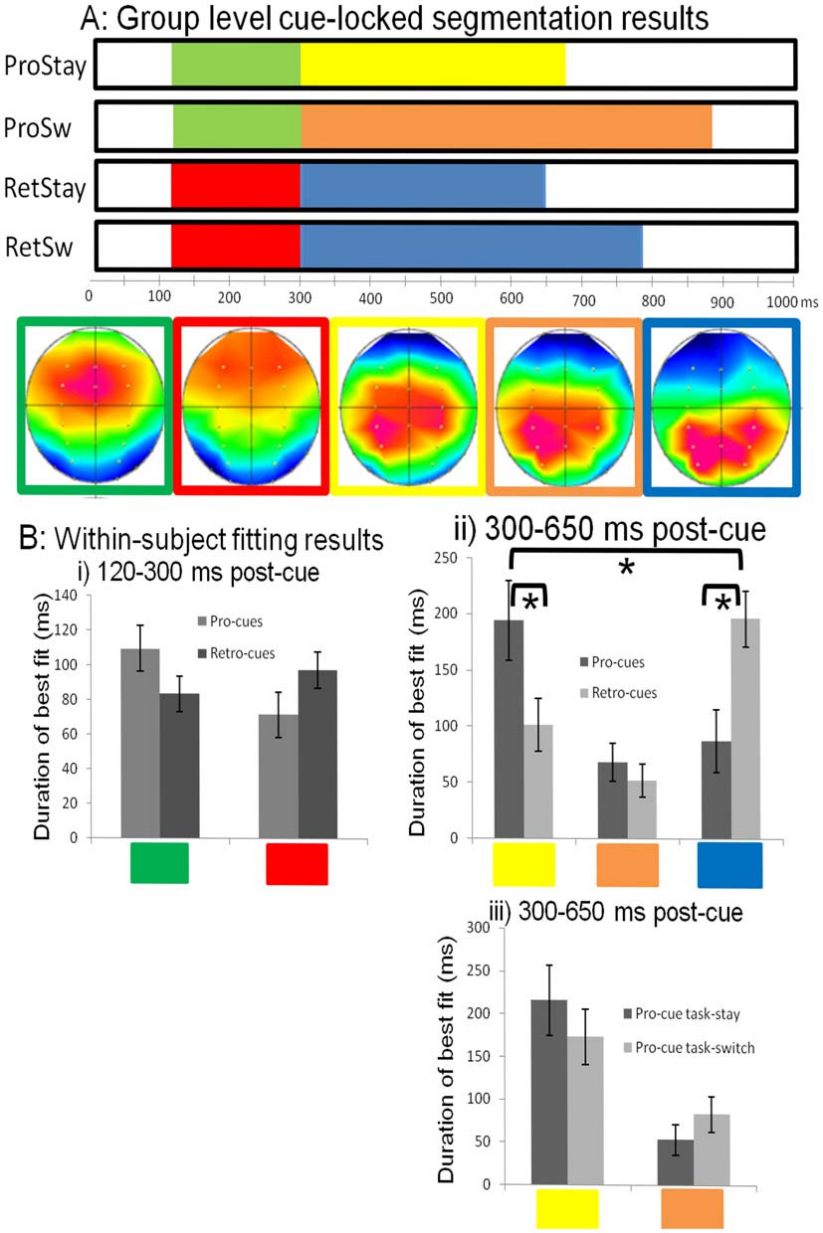
Topographical analyses. A) The result of the group-level segmentation process. The different colors show the durations of the various clusters of topography, across the four conditions, for the cue-locked epoch. Each of these clusters can be summarised as a single topography – the mathematical average of all topographies within that cluster – shown below. B) The results of the within-subject fitting procedure: i) the mean duration for which the ‘red’ and ‘green’ topographies were the best fit across pro-cue and retro-cue trials, between 120 and 300 ms; ii) the mean duration for which the ‘yellow’, ‘orange’ and ‘blue’ maps were the best fit across pro-cue and retro-cue trials, between 300 and 650 ms; and iii) the mean duration for which the ‘yellow’ and ‘orange’ maps were the best fit between 300 and 650 ms. In all cases the error bars show the standard error of the mean. In all cases the ‘*’ denotes significant differences, the absence of this denotes non-significant differences.

The best solution from our group-averaged segmentation explained over 92% of the variance in topographical distribution. The series of stable topographies that best explained the neural activity in the four experimental conditions can be seen in Figure 2A. Each trial type was characterised by a sustained positivity over the frontal areas (120–300 ms), followed by a sustained positivity over the parietal/occipital electrodes (300 ms onwards), which appeared to persist for longer on task-switch trials. Our segmentation result suggested that these topographies were different across the four conditions. The T-Anova confirmed the topographical differences identified by the segmentation: there was a significant topographical difference between pro-cue and retro-cue trials from 100 ms onwards; pro-cue stay and switch trials differed between 413–471, 515–560, and 591–650 ms. We also then used the candidate maps for the four trial-types for the within subject fitting procedure.

#### Within-participants fitting

We performed the fitting procedure between 120 and 300 ms, comparing the two candidate topographies across pro- and retro-cues. We used a repeated-measures ANOVA, with the within-participants factors of cue-type (pro- versus retro-cue), switching (stay versus switch) and topography (frequency of the ‘red’ map versus frequency of the ‘green’ map). The two-way interaction between cue-type and map only approached significance [F(1,13) = 3.673, p = 0.078]: the ‘green’ map was marginally more frequent than the ‘red’ map for pro-cue trials, and the ‘red’ map was marginally more frequent than the ‘green’ for retro-cue trials [ps = 0.078]. This topographical difference between pro-cues and retro-cues, between 120–300 ms, can be seen in Figure 2Bi. There was no significant interaction between switching and map [F(1,13) = 2.223, p = 0.160], or three-way interaction between switching, cue-type and map [F(1,13) = 0.051, p = 0.825].

We also performed the fitting procedure between 300 and 650 ms, comparing the three candidate maps across the four trial-types. This period is analogous to that of the parietal positivity observed in many other task-switching studies [14], [15], [18], [19], [20]–[23], [27], and evident in our own grand-average ERPs. Moreover, the broad centroparietal distribution in all of our topographies in this time window is similar to those observed in these other studies. Our repeated-measures ANOVA revealed a two-way interaction between cue-type and map [F(1,13) = 9.664, p = 0.003]. This resulted from the ‘yellow’ map being significantly more frequent on pro-cue trials than on retro-cue trials [p = 0.003], and the ‘blue’ map being significantly more frequent on retro-cue than pro-cue trials [p = 0.001]. The ‘orange’ map did not significantly distinguish pro- and retro-cues [p = 0.464]. This can be seen in Figure 2Bii. There was no interaction between switching and map [F(2,12) = 1.953, p = 0.184], and there were was no three-way interaction between switching, cue-type and map [F(2,12) = 1.341, p = 0.298]. Even though it was not justified by a significant three-way interaction, we also explicitly compared pro-cue stay and pro-cue switch trials, using the frequencies of the ‘yellow’ and ‘orange’ maps (see Figure 2Biii). This was done in order to establish whether there was a candidate topography that better accounted for switch than for stay trials, and which might thus reflect some TSR-like process. Our segmentation had suggested that, at a group level, pro-cue switch and cue task-stay trials could be distinguished on the basis of these two topographies, however the fitting result suggested that this was, at best, only marginally significant at a within-subject level [F(1,13) = 3.489, p = 0.084]. Moreover, this marginal result was primarily driven by the ‘yellow’ map being more frequent on pro-cue stay trials than on pro-cue switch trials [p = 0.075], rather than by the ‘orange’ map being more frequent on pro-cue *switch* trials than on pro-cue *stay* trials [p = 0.158]. In short, no candidate topography was a significantly better “fit” for switch than for stay trials.

#### Amplitude results

**Figure 3:**
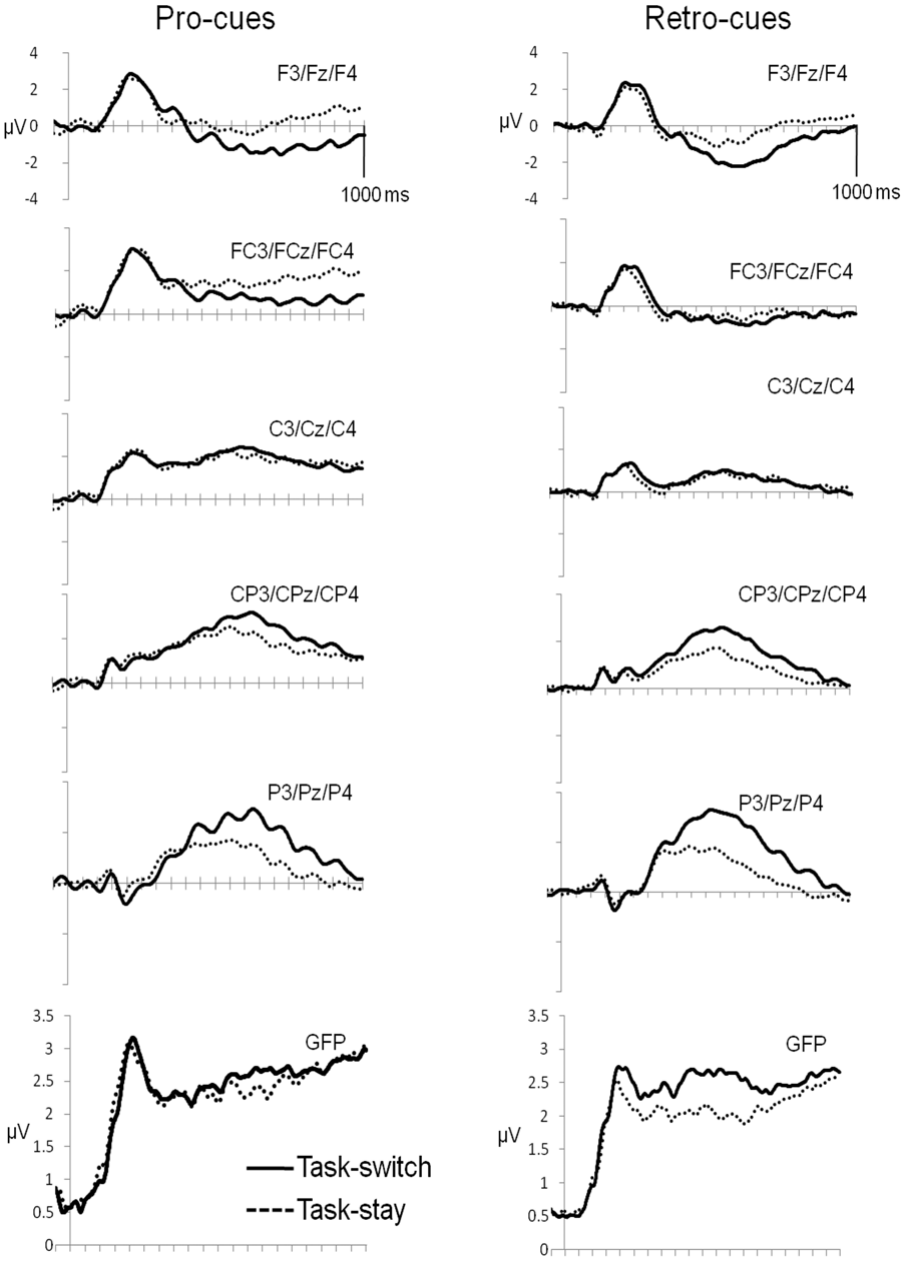
Grand-average waveforms. Grand-average waveforms, time-locked to the cue onset at 0 ms. These are shown separately for pro- (left-hand column) and retro-cue trials (right-hand column), for each recording site along the anteroposterior axis. Each waveform shown is the average of the left-, right-hemisphere and midline electrodes, with the solid lines representing task-switch trials and the dotted lines representing task-stay trials.

We also compared our four conditions using a more traditional amplitude analysis, to establish any differences between switch and stay trials, and whether these differed for pro- and retro-cues. These data can be seen in Figure 3. Whilst we found main effects of cue-type and switching, and interactions between each of these factors and one or both of our electrode factors, we only found a two-way interaction between switching and cue-type between 800–1000 ms [Fs>6.891, ps<0.021], resulting from a larger switch-repeat difference for pro-cues [ps<0.053] than retro-cues [ps>0.065], but this did not interact with electrode.

There was a significant main effect of cue-type, across consecutive bins from 120–480, 520–680, and from 880–960 ms [Fs>4.708, ps<0.049]. From 160–1000 ms cue-type interacted with electrode position along the anteroposterior axis [Fs>3.844, ps<0.039]. This was the result of pro-cues and retro-cues differing over the frontal, frontocentral and central electrode trios from 160–600 ms [ps<0.050], and over just the frontocentral and central electrode trios from 600–1000 ms [ps<0.059]. In all cases this was because pro-cue trials were more positive relative to retro-cue trials. Given the topographical differences that we observed between pro-cue and retro-cue trials, these interactions between cue-type and electrode position may have reflected primarily topographical differences.

Effects of switching started somewhat later than our cue-type effects; we also found an interaction between switching and electrode position along the anteroposterior axis, from 440–1000 ms [Fs>4.219, p<0.052]. This was the result of an initial switch-related positivity over the central, centroparietal and parietal trios [ps<0.031], from 400–480 ms, followed by a more sustained positivity over the centroparietal and parietal electrodes [ps<0.033] until 920 ms, and finally just over the parietal electrodes from 920–1000 ms [ps<0.031]. From 540–1000 ms this was accompanied by a switch-related negativity over the frontal electrodes [ps<0.036], which was occasionally present at the fronto-central electrodes [from 640–680, 720–760, and 880–920 ms, ps<0.041].

The GFP comparison also revealed cue-locked differences (these can be seen at the bottom of Figure 3). These were primarily between switch and stay trials, with switch trials having a greater GFP relative to stay trials (between 250 and 300 ms, and between 400 and 750 ms, [Fs>5.253, ps<0.039]). On occasion this interacted with cue-type (between 350 and 400 ms, 450 and 500 ms, and between 550 and 600 ms [Fs>5.581, ps<0.034]). In all cases this was because there was a significant switch versus stay GFP difference on retro-cue trials [ps<0.046], but no such difference on pro-cue trials [ps>0.454]. There was no main effect of cue-type on GFP.

## Discussion

We compared prospective and retrospective attentional set control. The behavioural data, both from the main experiment and the supplementary experiment, demonstrated that during the retro-cueing interval participants engaged in some time-consuming switch process. On short CTI trials, when less time was allowed for this process, there was a relative cost for switching attentional set. On long CTI trials, when enough time was allowed for this process, that cost had been overcome, or even slightly reversed. Our ERP analyses examined differences in topography, as well as changes in the amplitude of potentials, and GFP across the different conditions. Pro- and retro-cues could be distinguished on the basis of topography, presumably reflecting the recruitment of different neural mechanisms as participants maintain information in VSTM and select from it, versus prepare an attentional set for upcoming perceptual information. By contrast we were able to distinguish switch and stay trials in terms of the amplitude of potentials and GFP, but when controlling for these GFP differences the distribution of electrical activity was very similar.

### Applying an attentional set to remembered features

Pro- and retro-cues could be distinguished on the basis of topography, primarily that of a late parietal positivity (300–650 ms). The amplitude of the cue-locked effects also differed, with pro-cue trials being more positive than retro-cue trials over the frontal/central electrodes – though given the topographical result this ‘amplitude’ effect could have resulted either from amplitude or topographical differences.

There have been a number of recent electrophysiological studies that have explored the mechanisms by which features can be selectively stored in VSTM [e.g. 39], [40], though less is known about how features within objects can be *accessed* once stored. In particular, very few studies have contrasted the control required to select features in upcoming percepts (pro-cues) from the control required to select features in stored representations (retro-cues). However, previous experiments have explored the maintenance of the spatial location of objects and, in that context, Griffin and Nobre [3] found both early and late differences in ERP amplitudes between spatial retro- and pro-cues: an early positive non-lateralised potential over frontal scalp and a later increased relative positivity over posterior scalp regions, coupled with an increased negativity over the anterior scalp, for spatial retro-cues compared to pro-cues. In the current study retro-cue trials prompted the maintenance of object *features*, and thus the contrast between the pro-cue/retro-cue differences that we observed, and those previously observed [3] may depend on the feature-based nature of the selection mechanisms studied here. The differences between pro-cue and retro-cue ERPs in our feature-based paradigm were primarily evident in the topography of the parietal positivity typically seen in set-switching studies [e.g. 22], [23]. In particular, the differences that we observed likely stem from the fact that pro-cues allow for only partial preparation: following a pro-cue participants can prepare an attentional set (e.g. “attend color, but not shape”), but cannot prepare a stimulus-response mapping until the onset of the array (because the location of that particular colors and shapes changed on a trial-by-trial basis). By contrast, following a retro-cue, participants could both produce an attentional set *and* specify the stimulus-response mapping (e.g. “red object on the left and green object on the right”). Retro-cues enable this more complete preparation because the specific features for that trial are already known to the subject, being held in VSTM.

A previous study of feature selection within VSTM demonstrated that participants initially stored both task-relevant and task-irrelevant features, and then used spatially-specific attention mechanisms to distinguish the relevant from the irrelevant [2]. We suggest that a similar process is occurring here: participants store both the color and shape of the memory-array objects, then, following the onset of the retro-cue, they use attention mechanisms to select the relevant features in that particular memory-array. In this way retro-cues enable participants to prepare a specific stimulus-response mapping, something that pro-cues do not allow for, and we suggest that this underlies the topographical differences that we observed between these conditions. Recent imaging work has suggested that early visual cortices are recruited when maintaining information in VSTM [41], [42], perhaps explaining why the topography of our late parietal positivity is shifted more posteriorly, over the occipital electrodes, when participants are applying an attentional set to information held in VSTM.

### Distinguishing switch and stay trials

In the ERP data, switch trials were associated with a sustained late parietal positivity, and frontal negativity, relative to stay trials (from ∼400 ms onwards). The effect is akin to that labelled D-Pos [17]–[19], PP-AN [20] and the late parietal positivity and late frontal negativity described previously [22], [23], [25], [27]. In addition to replicating this electrophysiological effect, we also replicated the behavioural effect of reducing switch-costs with increasing amounts of preparation. Interestingly, participants prepared so well on set-switch trials that not only was the switch-cost fully overcome [see also 22]; [43] but it was even slightly reversed. It is particularly rare in the task-switching literature for participants to show a switch-related benefit. One possible explanation for our switch-related benefit is based on the finding that retro-cueing enables participants to access and sustain rapidly decaying VSTM representations [9]. On switch trials retro-cues require participants to re-access their stored representation of the array, to establish the locations of the previously unattended features. Revisiting of the stored memory array would not be necessary to the same extent on a repeat trial and, on switch trials, might result in a relative strengthening of the VSTM representation, and thus might facilitate a response being selected more rapidly upon probe presentation. Nonetheless, switching attentional set retrospectively, like switching attentional set prospectively [e.g. 15] was associated with some time-consuming process, which could be overcome prior to the onset of the imperative stimulus (i.e. the probe), and this control process was mirrored by a parietal positivity/frontal negativity in the electrophysiological data. It is important to note that one cannot test for this behavioural preparation effect in our pro-cue condition, as, even on short CTI pro-cue trials, participants could use the post-array interval, after the presentation of the array and preceding the probe, to which they responded, to engage in any time-consuming process.

It would be tempting to conclude that the time-consuming process inferred from the behavioural data is akin to TSR, and accordingly that the electrophysiological effects index this “mental gear change”-like process. However, in our data at least, this seems unlikely. Despite replicating the switch-stay parietal (and frontal) amplitude difference that others have reported, when taking into account amplitude differences (something that most studies have not done), switch and stay trials were more difficult to distinguish. This is particularly the case on retro-cue trials – precisely those trials for which we are able to evidence behaviourally some time-consuming switch process. Indeed retro-cue trials presented the best chance of demonstrating some TSR-like process, because participants can both produce an attentional set and fully specify a stimulus-response mapping following these types of cue. However, it was *especially* in this case that our group-segmentation procedure could not distinguish switch and stay trials on the basis of normalised topography. Despite setting the criteria for distinguishing topographies particularly high, separating any topographies correlated by <0.97, we were unable to find any topography associated with switch that was not also present on non-switch trials. We would therefore suggest that the switch-stay parietal difference, typically observed in set-switching paradigms and replicated here, is primarily the result of a substantial amplitude difference, with only very subtle differences in the configuration of neural generators, if at all. On this basis, at least in our own data, this particular component does not seem to fit the pattern we would expect for a TSR-like process, which, by definition, would occur on switch but not stay trials and therefore should engage a distinct set of neural generators (or at the least a difference in the weighting of activity across those same generators). Wylie and colleagues came to the same conclusion through a very similar topographical analysis [36]. Interestingly the paradigm used by Wylie and colleagues was very different to that used here: participants switched between letter and number judgment tasks, only pro-cues were used, task-cues rather than transition-cues were used, and CTI was not varied in the same way. However, the result was remarkably similar: the behavioural data reflected a switch cost that was overcome with preparation; in the ERP data, despite a large GFP difference, the normalised distribution of effects on switch and stay trials were statistically indistinguishable. Thus, despite differences in experimental paradigm, converging findings were obtained: switch and stay trials recruit a qualitatively similar set of neural generators deployed to a quantitatively different extent. In turn, this is more indicative of the application of a competitive bias for attentional set selection, rather than of stimulus-response reconfiguration per se.

Despite replicating the large switch/stay parietal amplitude difference, we were also unable to differentiate significantly the pro-cue switch and pro-cue stay trials on the basis of normalised topography; any differences that we observed at a group segmentation level were not statistically significant at a within participant level. That said, it would be unwise to use these data to conclude that the amplitude effect that we observed in the pro-cue trials does not index some TSR-like process, since we were unable to evidence such a process behaviourally in this condition. Instead we can firmly draw our conclusions from the retro-cue trials, for which we can incorporate all three pieces of evidence: the behavioural evidence for a cue-locked time-consuming switching process, the amplitude effect and the topographical analyses.

As is the case with our retro-cue trials, some previous studies have also suggested that their observed parietal positivity (and frontal negativity) does not index an additional obligatory switch-related process, despite mirroring a reduction in switch-costs with preparation: for example Astle, Jackson and Swainson [23] recently demonstrated that it is possible to switch task *without* this process ever having happened. When participants were supplied with salient spatial information with which to distinguish which task they were performing, the cue-locked parietal positivity (and accompanying frontal negativity) was absent. By contrast both were very much present in a condition in which participants switched between the same tasks but without this spatial information. This would seem to preclude the possibility that the ERP effect observed by Astle et al. [23] indexed stimulus-response reconfiguration specifically – both conditions used by Astle et al. had identical stimulus-response mappings, and had a similar degree of switch-cost reduction with preparation, whereas only one had a parietal positivity. It would also suggest that whatever the cue-locked parietal positivity indexed in that study, it was not strictly necessary for switching task – one could switch tasks without it provided that task selection was supported by appropriate spatial information.

Despite typically mirroring the reduction in switch-cost with preparation, the specific functional role of the cue-locked parietal positivity and frontal negativity remains unclear. Whilst it is likely to index some active process [29], rather than simply the passive dissipation of the previously performed task-set [30], we are of the view this is more likely to take the form of a top-down attentional bias that operates similarly in switch and stay trials, though to differing extents. Top-down attentional signals are thought to bias neural processes throughout multiple levels of processing, according to, say, expected location, or object features that are task-relevant [10]. In relation to our own data, rather than the switch-related parietal positivity indexing some ‘mental gear change’, or set reconfiguration (although this is not to say that such processes do not occur), it might reflect the relative biasing of those task-relevant features necessary for a particular task. Most importantly, the same process might reasonably occur on stay trials as well as on switch trials; however, because those features were already partially biased from the preceding trial, this might not happen with the same power or for the same duration on stay trials [as in the present study; see also 36].
